# Field evaluation of ethnomedicinal plants’ smoke knockdown and repellent efficacy against pre-bedtime biting malaria vectors in Mazowe district, Zimbabwe

**DOI:** 10.1371/journal.pone.0336943

**Published:** 2025-11-14

**Authors:** David Singleton Nyasvisvo, Shadreck Sande, Rudo Sithole, Tamuka Nhiwatiwa

**Affiliations:** 1 Department of Biological Sciences and Ecology, University of Zimbabwe, Mt. Pleasant, Harare, Zimbabwe; 2 Department of Fisheries and Ocean Sciences, University of Namibia, Henties Bay, Namibia; Universidade Federal do Para, BRAZIL

## Abstract

The study investigated the knockdown and repellent efficacy of smoke from native plants, *Vitex payos* (Lour.) Merr., *Peltophorum africanum* Sond., and *Adansonia digitata* L., against malaria vectors in Bare ward, Mazowe district, Zimbabwe. Three to five-day-old *An. gambiae* sensu lato (s.l.) were exposed to smoke from the three plants over 150 minutes during knockdown tests. Mortality was scored after 24 hours. A randomized 4 x 4 Latin square design was used to assign plant smoke and control treatments to huts and nights during repellency tests. Repellency was estimated as percentage reduction in biting using CDC light traps between 1800 and 2200 hours. Burning charcoal without plant material was used as control. SPSS software was used for data analysis. KdT_50_ and kdT_90_ rates were determined using probit analysis. A negative binomial GLM with a log link function and an emmeans post-hoc test was used to model the number of *An. funestus* s.l. caught based on smoke treatment, night and hut. *Vitex payos* and *P. africanum* knocked down 100% of the *An. gambiae* s.l. within the 150-minute exposure period. The kdT_50_ and kdT_90_ rates were more rapid for *V. payos* (37.8 and 69.7 min) and *P. africanum* (44.8 and 89.7 min) than for *A. digitata* (77.8 and 146.9 min). The percent 24-hr mortality was 91.7 (*V. payos*)*,* 80 (*P. africanum*) *and 71.6* (*A. digitata*)*.* Smoke from *V. payos* (86%, p = 0.008) and *P. africanum* (73%, p = 0.028) significantly reduced *An. funestus* s.l. biting. Smoke from *V. payos* and *P. africanum* merits further investigation since it has the potential to provide an additive benefit to ITNs by targeting proportions of malaria vectors that bite before bedtime. Studies to determine whether the use of smoke from these plants has a community effect that can lead to the reduction of malaria burden are required and significant.

## Introduction

The major malaria vector control methods in Zimbabwe are insecticide-based indoor residual spraying (IRS) and insecticide-treated bed nets (ITNs), complemented with larviciding [1]. The primary malaria vectors in Zimbabwe belong to the *Anopheles gambiae* and *An. funestus* complexes [1,2]. Mostly they bite and rest indoors with biting peaks late at night and in the early hours of the morning and hence are vulnerable to control using IRS and ITNs [3]. However, changes in mosquito behaviour such as early and late indoor biting as well as outdoor biting and resting decrease the effectiveness of ITNs and IRS to prevent malaria transmission [4]. It appears little attention is paid to malaria vectors that bite during periods when people are not under protection from ITNs in Zimbabwe. As reported elsewhere globally [5], the country is also faced with growing threats of insecticide resistance and residual malaria transmission. This makes the search for alternative and/or complementary tools for the control of local malaria vectors such as mosquito repellents a priority.

The use of mosquito repellents to avoid human-vector contact is well documented [6–8] and their use in preventing systemic infections constitutes a fundamental public health effort [9,10]. Conventional synthetic repellents such as N,N-diethyl-m-toluamide (DEET) are used for protection prior to bedtime and are effective in diverting opportunistic vectors to alternative hosts [9]. However, malaria tends to predominate in low-income communities where access to DEET and other synthetic repellent products is limited by scarcity and affordability challenges [11–13]. Coupled with this, conventional synthetic repellents are associated with negative effects related to neurotoxicity and dermatitis [14] and reduced efficacy due to sweating and allergic reactions [15]. Communities globally establish alternative, cost-effective and unconventional means of vector control and home remedies of unknown efficacy in situations where conventional methods are unsuccessful, unaffordable or unavailable [8,16]. The repellent properties of plants to mosquitoes were known before the advent of synthetic chemicals [7,13,17,18]. The use of native plants for repelling mosquitoes is widespread in many communities and very common across different cultures [13,19–25].

Burning or smoking repellent plants is a common method rooted in traditional practices used for immediate and localized mosquito control by communities in malaria-endemic parts of Zimbabwe [26,27] and throughout the world [8,9,13,28]. Furthermore, there are several reports where smoke produced from burning a variety of plants was effective in reducing biting by various *Anopheles* species [29–33]. The use of effective native plants in the control of local malaria vectors could be valuable and sustainable option in establishing integrated malaria vector control in Zimbabwe since the native plants are readily available and the methods employed are more economical, culturally acceptable and simple. However, despite the widespread usage of plants as repellents by rural communities in malaria-endemic parts of the country, there is lack of scientific data to confirm their efficacy and consequently plants have not gained significant traction in vector control programs in Zimbabwe, particularly in Bare ward of Mazowe district.

Bare ward is one of the malaria-endemic wards in Mazowe district where ITNs are the main intervention tool. However, despite the high coverage of ITNs, malaria remains a major public health problem in this ward [27], suggesting that the existing malaria vector control interventions are insufficient to curb residual malaria transmission in this ward. *Vitex payos*, *P. africanum*, and *A. digitata*, are native plants indigenous to Zimbabwe with recognition for their purported localized mosquito control through burning by communities in Bare ward [27]. While there are numerous studies on plant smoke repellency across Africa [29–33], the studies did not focus on *V. payos*, *P. africanum* and *A. digitata* and there is no documented evidence relating to the repellent efficacy of these plants against local malaria vectors. Multiple phytochemicals with literature-confirmed mosquitocidal properties were identified from methanol extracts of *V. payos*, *P. africanum* and *A. digitata* [34]. However, it appeared the efficacy of these plants against mosquito bites had not been investigated in scientific experiments and warranted further research.

Community involvement and biocultural rights play a crucial role in the development of novel mosquito repellents that are not only effective but also sustainable, culturally relevant and equitable. The scientific evaluation of the repellent efficacy of *V. payos*, *P. africanum* and *A. digitata* substantiates claims of their efficacy and validates or confirms their effectiveness for the rationalization of their use against mosquito bites. It is crucial for the development of vector control tools that are culturally acceptable and accessible for communities that live in malaria-endemic parts of the country. Scientific evaluation is also important for the identification and isolation of bioactive phytochemical compounds that can be used in the development of novel plant-based repellents. A direct demonstration of usability and value of native plants and indigenous technologies to prevent malaria increases local communities’ confidence and is also important for planning conservation programs that are targeted at plants of biomedical value. In the present study, the comparative knockdown, killing and repellent efficacy of smoke produced from the thermal expulsion of native plants *V. payos*, *P. africanum* and *A. digitata*, were evaluated under natural field conditions at Chawasarira village in Bare ward, Mazowe district, Zimbabwe. It was hypothesized that smoke from the three ethnomedicinal plants would (a) suppress pre-bedtime biting rates of *Anopheles* mosquitoes by at least 75% and (b) produce ≥90% 24-hr mortality, thus outperforming charcoal smoke control huts and meeting Environmental Protection Agency (EPA) (2022) and World Health Organization (WHO) (2013b) efficacy thresholds/minimum performance standards for spatial repellents.

## Materials and methods

### Study area

Field studies were conducted at Chawasarira village (Four-stream area) (16.91°S, 31.07°E; elevation 1322m), Bare ward in Mazowe district. Mazowe district, with 35 administrative wards, is a rural district in Mashonaland Central province. Falling under agroecological region II, the district covers an area of approximately 1 444km^2^, with an estimated population of 0.29 million [35]. Bare is one of the wards with a high incidence of malaria in Mazowe district [36]. It comprises 17 villages and 1 571 households which are randomly distributed. Chawasarira village has the highest incidence of malaria in Bare ward. It is accessible, and has ecological conditions that provide potential breeding habitats and survival of both *Anopheles gambiae* and *An. funestus* complexes [36]. Insecticide-treated bed nets are the main vector control tool used for protection against malaria infection at Chawasarira village.

### Study period

The study was conducted in April and May 2025, a period that represents a transition between the wet and dry seasons and part of the peak malaria transmission period in the study area. The peak malaria incidence occurs between March and May following the main rainfall season of November to March [37,38].

### Selection and general description of test plants

*Vitex payos*, *P. africanum*, and *A. digitata* were identified through an ethnobotanical survey conducted in Mazowe and Shamva districts [27]. The three plants were selected for field efficacy studies because they are available, accessible in abundance and have a history of safe traditional use. In addition, they are native to Zimbabwe and are among the plants most commonly used for protection against mosquito bites in the study area but without published data on their repellent efficacy against the main malaria vectors in the country. Communities use the plants alone early in the evening before retreating to ITNs [27]. To our knowledge, besides the identification of multiple phytochemicals with literature-confirmed mosquitocidal properties, there were no previous local records on the scientific evaluation of the mosquito repellent properties of *V. payos*, *P. africanum* and *A. digitata*.

*Vitex payos* (common names: Mutsubvu or chocolate berry; Family: Lamiaceae) is a common tree or shrub that can reach a height of 8m and has a widespread distribution in Zimbabwe [39]. It has many ethnomedicinal uses [40]. Bioactive compounds from *V. payos* inhibit the growth of mosquito larvae [41,42]. *Peltophorum africanum* (common names: Muzeze or Rhodesian blackwood; Family: Fabaceae) is a deciduous tree that can grow up to 10m in height, with a spreading crown, and is widespread in Zimbabwe and in other southern African countries [43]. *Peltophorum africanum* has many ethnomedicinal and ethnoveterinary uses [40,44,45]. *Adansonia digitata* (common names: Muwuyu or baobab tree; Family: Malvaceae) is a spreading tree that grows up to 15m in height, is native to Zimbabwe, and has a widespread distribution throughout the hot and dry regions of tropical Africa [39,46]. Culturally, *A. digitata* is a multipurpose tree that offers protection and provides food, clothing, medicine, and raw materials for many items [47].

### Collection, identification and preparations of the test plants

Plant samples were collected from Chawasaria village, Bare ward in Mazowe district, and processed following relevant national norms and legislation and according to standard protocols provided by the National Herbarium and Botanic Garden, Harare, Zimbabwe. They were identified by a taxonomist and authenticated at the National Herbarium and Botanic Garden, Harare, Zimbabwe, where voucher specimens were deposited for records and future reference purposes. *Vitex payos* leaves, *P. africanum* leaves as well as *A. digitata* fruit shells were carefully cleaned using tap (borehole) water. The samples were dried in the shade under similar temperature (range 20–25°C) and relative humidity (range 50–60%) conditions until dry weight stabilized. The dried samples were ground using a Christy and Norris Junior laboratory mill to produce fine particles of uniform size across all the samples. The powders from each plant species were kept separately in airtight containers until they were made available for use in field experiments.

### Selection of huts for knockdown and repellency bioassays

Four (3 experimental and 1 control) traditional-style (mud-walled grass-thatched) round huts used as kitchens were randomly selected from the Four-stream area under Chawasarira village in Bare ward for use in knockdown tests. An additional four (3 experimental and 1 control) traditional-style (mud-walled grass-thatched) rectangular huts with open eaves and unscreened small holes used as sleeping rooms were randomly selected for the repellency tests. Open eaves and unscreened holes allow free entry and exit of mosquitoes into the huts. To minimize the variation and to burn the same amount of plant material for smoking, all the huts selected for each activity were similar in type and approximately of the same size. Kitchens had an average wall height of 2m and a diameter of 4m while sleeping huts had an average floor plan area of 3m x 3m and height of 2m. All the selected huts were accessible and had a minimum distance of 200m from each other to avoid the diversion of smoke from one hut to the next. In addition, all the huts were free from residual insecticides used for malaria control since the National Malaria Control Program (NMCP) does not conduct IRS in Mazowe district.

### Rearing of *An. gambiae* s.l.

*Anopheles* mosquitoes were collected as larvae and pupae from a breeding site (16.86°S, 31.09°E; elevation 1320m) located at Timuri village in Bare ward, Mazowe district. The larvae and pupae were reared to adults at Bare Rural Health Centre’s temporary insectary following recommended techniques for rearing mosquitoes as described by Das et al. [48]. The emerged adults were provided with a 10% sugar solution, identified using morphological identification keys [49] and only the female *An. gambiae* s.l. were selected for use in the knockdown tests.

### Knockdown bioassays and scoring 24hr mortality rates

Thermal expulsion of individual plant materials was implemented in both knockdown and repellency tests using modified traditional charcoal “*mbaura*” stoves with fixed metal cups for burning repellent plant material, although regular mbaura stoves can also be used with a removable metal plate/cup placed on top for the thermal expulsion process. Knockdown activity tests were conducted in the morning between 0800hrs and 1100hrs and preparations of the rooms included removing all people and animals (where applicable) from the kitchen huts. The experimental huts were fumigated for 150 minutes by placing 50g of plant powder on a thin metal cup fixed on the traditional “*mbaura*” stove with burning charcoal at the centre and original fireplace of each hut following methods described by Lukwa et al. [50]. The control was conducted under the same conditions except that the control hut had burning charcoal only without the repellent plant materials under evaluation.

Twenty, 3–5-day old female *An. gambiae* s.l. mosquitoes were placed in a clean paper cup that was covered using a piece of white clean mosquito netting. Each mosquito netting had a small opening for introducing mosquitoes using a mouth aspirator and the opening was kept closed using clean cotton wool throughout the experiments. The paper cups were uniformly placed in each fumigated hut at a height of 1.2 m and a distance of 1.5 m from the *mbaura* stove with burning plant material. Due to the limited number of female *An. gambiae* s.l. available for the tests, only three paper cups (pseudo-replicates) were used for each treatment. The number of knocked down mosquitoes in each paper cup was recorded after every 10 minutes for a 150-minute exposure period [50]. Knocked down mosquitoes were defined as those that could no longer maintain normal posture, lying on their backs and unable to fly [51]. After 150 minutes, all the paper cups were collected; the mosquitoes were transferred to clean well-labelled paper cups, and fed using a 10% sugar solution sprinkled on cotton wool. The paper cups from each treatment were kept separately in cooler boxes for a 24-hour holding period before mortality was scored. Mortality in each paper cup was recorded after 24 hours by counting all the dead mosquitoes and percent mortality was calculated for each treatment. Temperature and humidity were recorded using a digital thermohygrometer during both the exposure and 24-hr holding periods. Live mosquitoes from the tests were killed by freezing.

### Smoke repellency tests

To minimize bias due to the variation in hut location and different sampling nights, a 4x4 Latin square design (LSD) was used to randomly assign treatments to experimental huts and treatment nights [52]. Three replicated Latin squares were completed; hence, the experiments were conducted over 12 nights, with the four treatments rotated amongst four huts (3 replicates with 4 treatment nights per replicate). Rotation between the experimental huts was such that every treatment was tested in every hut an equal number of times. Similar to the knockdown tests, the control huts had the same conditions but with burning charcoal only without plant material. Battery-powered (6V) Centers for Disease Control (CDC) light traps were used to collect indoor host-seeking mosquitoes [9,53,54]. The CDC light traps were operated from 1800hrs to 2200hrs. The experimental huts were fumigated by placing 50g of plant powder in a thin metal cup on the traditional charcoal “*mbaura*” stove with burning charcoal every hour between 1800 hours and 2200 hours. Trap collection bags were collected at 2200 hrs to evaluate the impact of the fumigation of individual huts on mosquito catches.

One smoke-free intervening day was left in between the experiments to clean and ventilate the experimental huts and to remove contamination from previous treatments due to residual effect of smoke [51]. In addition, CDC light traps were cleaned using soap and water to avoid the residual effect of treatment on the next sampling night. The repellent efficacy of smoke was obtained by comparing the number of mosquitoes caught host-seeking in huts with the treatment (a traditional repellent plant smoke) with those caught in the control (charcoal smoke) [19,33].

### Morphological identification and processing of *Anopheles* mosquito specimens

Female *Anopheles* mosquitoes were separated from the males and identified using the morphological keys [49]. There was no DNA extracted from the mosquitoes for PCR analysis due to logistical constraints.

### Statistical analyses

Microsoft Excel was used for data entry and cleaning. The clean MS Excel file was imported into IBM Statistical Package for Social Sciences (SPSS) version 29.0 for data analysis**.** Probit analysis was used to determine the time required to knockdown 50% and 90% (kdT_50_ and kdT_90_) of the mosquitoes [55]. Ninety (90) percent 24-hr mortality was used as the minimum performance standard for insecticidal effect while 75% was used as the minimum performance standard for spatial repellency [56].

A generalized linear model (GLM) with a negative binomial distribution and a log link function was used to model the number of *An. funestus* s.l. and *Culex* spp mosquitoes caught based on plant smoke treatment, hut and night. Pairwise comparisons were made with effects observed between the plant smoke and control groups using the estimated marginal means (emmeans) post-hoc test. Least significant difference (LSD) was specified as the adjustment for multiple comparisons; p ≤ 0.05 was considered significant.

The repellent effect of the smoke was determined as percentage reduction in biting calculated as follows:


$$𝐏𝐑\;=\;\frac{𝐍𝐂-𝐍𝐓}{𝐍𝐂}\times \text{100},$$

where **PR** is the percentage reduction in biting, **NC** is the number of mosquitoes caught in control and **NT** is the number of mosquitoes caught in treatment [19,33]. Mean percentage reductions and their 95% confidence intervals were calculated for each species and treatment.

### Ethics statement

The study did not involve human participants, no personal data was collected, and no invasive procedures were performed on humans or animals. The study complied with all relevant regulations. The investigation protocols were approved by the Department of Biological Sciences and Ecology, University of Zimbabwe, Harare, Zimbabwe. Field site access/permission to conduct the study in Mazowe district was approved by Dr. Clement Tshuma, Provincial Medical Director (PMD), Mashonaland Central Province, Ministry of Health and Child Care (MoHCC), Zimbabwe. Local researchers under the Department of Biological Sciences and Ecology, University of Zimbabwe, Harare, Zimbabwe, conducted the study and a separate field permit was not required for research conducted within the country. However, collaboration with local authorities and traditional leaders was required to ensure that the research was conducted with the necessary permissions and approvals. Entry into the community for the field studies was organized with the assistance of Mrs. Rosemary Chibatamoto, District Environmental Health Officer (DEHO) for Mazowe district, MoHCC, Zimbabwe. Verbal consent, which is more inclusive, ethically preferable and better suited to creating rapport with the community [57], was sought and obtained from traditional leaders. Informed written consent was sought and obtained from all heads of households involved in the study. All experiments were performed by the investigators and trained MoHCC Environmental Health Technicians (EHTs) based at Bare Rural Health Centre. All relevant national guidelines and international protocols were followed to ensure the safety, privacy, confidentiality and dignity of the households involved.

## Results

### Comparison of mosquito knockdown effect of three different repellent plant species

The smoke from burning *V. payos*, and *P. africanum* knocked down 100% of the *An. gambiae* s.l. mosquitoes whilst that from *A. digitata* knocked down 85% of the *An. gambiae* s.l. within the 150-minute exposure period as opposed to zero knockdown effect from the charcoal control (Table 1).

**Table 1:** Comparison of mosquito knockdown effect of smoke produced from burning different repellent plant materials at Bare ward, Mazowe district, Zimbabwe.

Exposure Time (min)	Percentage mosquito knockdown			
	*V. payos*	*P. africanum*	*A. digitata*	Control
0	0	0	0	0
10	3	0	0	0
20	18	12	5	0
30	25	30	15	0
40	42	42	28	0
50	58	52	40	0
60	80	65	55	0
70	92	70	62	0
80	100	80	58	0
90	100	85	65	0
100	100	95	68	0
110	100	100	70	0
120	100	100	75	0
130	100	100	78	0
140	100	100	80	0
150	100	100	85	0

### KdT_50_, kdT_90_ values and 24-hr mortality rates

The knockdown effect of smoke produced from thermal expulsion of the three native plants determined over a 150-minute period indicated that kdT_50_ and kdT_90_ (knockdown rates) were more rapid for *V. payos* and *P. africanum* than for *A. digitata* (Table 2). Smoke from thermal expulsion of the three plants caused pronounced mortality on adult female *An. gambiae* s.l. after the 24-hr holding period, although none of the plant smoke achieved a 100% 24-hr mortality. Smoke from *V. payos* achieved 91.7% 24-hr mortality, which is higher than the 90% 24-hr mortality that was set as minimum criteria for insecticidal effect. There were no *An. gambiae* s.l. mortalities recorded in the control after the 24-hr holding period.

**Table 2:** kdT_50_, kdT_90_ and 24-hr mortality of *An. gambiae* s.l. due to smoke produced from burning different repellent plant materials at Bare ward, Mazowe district, Zimbabwe.

Smoke treatment	kdT_50_ (min) (95% confidence interval)	kdT_90_ (min) (95% confidence interval)	Percent 24-hr mortality (95% confidence interval)
*V. payos*	37.89 (31.99; 43.51)	69.74 (59.97; 86.04)	91.67 (72.69; 110.64)
*P. africanum*	44.75 (41.60; 47.79)	89.66 (83.18; 97.85)	80 (67.58; 92.42)
A. *digitata*	77.78 (68.02; 87.49)	146.95 (138.20; 170.11)	71.67 (64.50; 78.84)
Control	–	–	0

### Abundance of female mosquitoes collected during plant smoke repellency tests

Overall, 174 and 131 female *Anopheles* and *Culex* mosquitoes respectively were collected over 12 treatment nights (Table 3). The female *Anopheles* were morphologically identified as *An. funestus* s.l. (56.9%), *An. pretoriensis* (30.5%), *An. gambiae* s.l. (10.3%) and *An. rufipes* (2.3%). The distribution of female *Anopheles* mosquitoes by treatment were as follows: control (56.3%), *A. digitata* (17.8%), *P. africanum* (16.7%) and *V. payos* (9.2%).

**Table 3:** Number of female *Anopheles* and *Culex* mosquitoes caught during smoke repellency tests at Bare ward, Mazowe district, Zimbabwe.

Smoke treatment	Mosquito species				
	*An. funestus* s.l.	*An. gambiae* s.l.	*An. pretoriensis*	*An. rufipes*	*Culex*
*V. payos*	8	2	6	0	12
*P. africanum*	15	3	10	1	22
*A. digitata*	20	2	8	1	19
Control	56	11	29	2	78
Total	99	18	53	4	131
Mean ± SE_mean_	2.06 ± 1.75	0.38 ± 0.61	1.10 ± 1.04	0.08 ± 0.28	2.73 ± 2.46

### Association between plant smoke and *An. funestus* s.l. catches

*Anopheles funestus* s.l. data showed overdispersion (variance greater than mean) while the samples sizes for the other *Anopheles* species were very small. The negative binomial generalized linear model fit the data well, as indicated by the goodness-of-fit statistics: Deviance/df = 0.215, Pearson Chi-Square/df = 0.165, AIC = 188.700. The Omnibus test was significant, X^2^(9) = 17.018, p = 0.048, indicating that the model provided a better fit to the data compared to the intercept-only model. Regarding the individual predictors, smoke treatment [X^2^(3) = 14.725, p = 0.002] was a significant predictor of the *An. funestus* s.l. catches. In contrast, hut [X^2^(3) = 0.618, p = 0.892] and night [X^2^(3) = 0.457, p = 0.928] did not have a significant effect on the number of *An. funestus* s.l. caught. The smoke treatment significantly affected the number of *An. funestus* s.l. caught while hut and night did not significantly affect the number of *An. funestus* s.l. caught. The emmeans post-hoc test showed that *An. funestus* s.l. catches based on smoke from thermal expulsion of *Vitex payos* (p = 0.008) and *P. africanum* (p = 0.028) were significantly lower than the control while those from smoke from thermal expulsion of *A. digitata* were not significantly different from the control (p = 0.063). There were no significant differences on *An. funestus* s.l. catches from the three plants’ smoke (Table 4).

**Table 4:** Pairwise comparisons of estimated marginal means based on *An. funestus* s.l. catches.

Smoke treatment		Mean difference	Standard error	d.f.	P-value	95% confidence interval for difference
*A. digitata*	Control	−2.9685	1.59583	1	0.063	(−6.0962; 0.1593)
	*P. africanum*	0.4308	0.77595	1	0.579	(−1.0900; 1.9516)
	*V. payos*	1.0194	0.68124	1	0.135	(−0.3158; 2.3546)
Control	*A. digitata*	*2.9685*	1.59583	1	0.063	(−0.1593; 6.0962)
	*P. africanum*	3.3993^a^	1.54993	1	**0.028**	(0.3615; 6.4371)
	*V. payos*	3.9879^a^	1.50369	1	**0.008**	(1.0407; 6.9351)
*P. africanum*	*A. digitata*	−0.4308	0.77595	1	0.579	(−1.9516; 1.0900)
	Control	−3.3993^a^	1.54993	1	**0.028**	(−6.4371; −0.3615)
	*V. payos*	0.5886	0.56263	1	0.295	(−0.5141; 1.6913)
*V. payos*	*A. digitata*	−1.0194	0.68124	1	0.135	(−2.3546; 0.3158)
	Control	−3.9879^a^	1.50369	1	**0.008**	(−6.9351; −1.0407)
	*P. africanum*	−0.5886	0.56263	1	0.295	(−1.6913; 0.5141)

^a^. The mean difference is significant at the 0.05 level.

### Association between plant smoke and *Culex* mosquito catches

*Culex* mosquito data showed overdispersion. The negative binomial generalized linear model fit the data well, as indicated by the goodness-of-fit statistics: Deviance/df = 0.278, Pearson Chi-Square/df = 0.178, AIC = 209.108. The Omnibus test was significant, X^2^(9) = 19.040, p = 0.025, indicating that the model provided a better fit to the data compared to the intercept-only model. Regarding the individual predictors, smoke treatment [X^2^(3) = 16.610, p = 0.001] was a significant predictor of the *Culex* mosquito catches. In contrast, hut [X^2^(3) = 0.187, p = 0.980] and night [X^2^(3) = 0.244, p = 0.970] did not have a significant effect on the number of *Culex* mosquitoes caught. These results suggest that smoke treatment significantly affected the number of *Culex* mosquitoes caught while hut and night did not significantly affect the number of Culicine mosquitoes caught. The emmeans post-hoc test showed that *Culex* mosquito catches based on smoke from thermal expulsion of *V. payos* (p = 0.008), *P. africanum* (p = 0.028) and *A. digitata* (p = 0.019) were significantly lower than the control. There were no significant differences on the number of *Culex* mosquitoes caught using each of the three plants’ smoke (Table 5).

**Table 5:** Pairwise comparisons of estimated marginal means based on *Culex* mosquito catches.

Smoke treatment		Mean difference	Standard error	d.f.	P-value	95% confidence interval for difference
*A. digitata*	Control	−4.9155^a^	2.08839	1	**0.019**	(−9.0086; −0.8223)
	*P. africanum*	−0.2802	0.87567	1	0.749	(−1.9965; 1.4360)
	*V. payos*	0.5428	0.70807	1	0.443	(−0.8450; 1.9306)
Control	*A. digitata*	4.9155^a^	2.08839	1	**0.019**	(0.8223; 9.0086)
	*P. africanum*	4.6352^a^	2.11366	1	**0.028**	(0.4925; 8.7779)
	*V. payos*	5.4582^a^	2.05154	1	**0.008**	(1.4373; 9.4792)
*P. africanum*	*A. digitata*	0.2802	0.87567	1	0.749	(−1.4360; 1.9965)
	Control	−4.6352^a^	2.11366	1	**0.028**	(−8.7779; −0.4925)
	*V. payos*	0.8230	0.77921	1	0.291	(−0.7042; 2.3502)
*V. payos*	*A. digitata*	−0.5428	0.70807	1	0.443	(−1.9306; 0.8450)
	Control	−5.4582^a^	2.05154	1	**0.008**	(−9.4792; −1.4373)
	*P. africanum*	−0.8230	0.77921	1	0.291	(−2.3502; 0.7042)

^a^. The mean difference is significant at the 0.05 level.

### Percentage reduction in biting

The three individual plant powders exhibited significant percentage reduction in biting by the different species of mosquitoes, the degree of repellency of the smoke varied depending on the species of the plant, and the species of the mosquito (Table 6). Percentage reduction in biting by smoke from thermal expulsion of *V. payos* was above 75% for all the mosquito species; *A. digitata* for *An. gambiae* s.l. and *Culex* spp; and below 75% for *P. africanum* for all the mosquito species (Table 6).

**Table 6:** Mean percentage reduction in biting, with 95% confidence interval, due to smoke produced from burning different repellent plant materials at Bare ward, Mazowe district, Zimbabwe.

Smoke treatment	Mean percentage reduction in biting (95% confidence interval)				
	*An. funestus* s.l.	*An. gambiae* s.l.	*An. pretoriensis*	*An. rufipes*	*Culex*
*V. payos*	85.7 (73.6; 97.0)	81.8 (40.5; 124)	75.9 (34.3; 119)	100 (100; 100)	84.6 (70.8; 91.4
*P. africanum*	73.2 (52.3; 88.1)	72.7 (22.3; 129)	65.1 (49.8; 77.2)	50 (−126; 226)	72 (61.5; 82.5)
*A. digitata*	64.3 (44.7; 82.5)	81.8 (30.0; 126)	72.4 (10.3; 128)	50 (−126; 226)	75.6 (61.4; 84.6)

## Discussion

The knockdown and repellent efficacy of smoke generated by thermally expelling three native plants, *V. payos*, *P. africanum*, and *A. digitata,* were investigated under field conditions in Mazowe district, Zimbabwe. Trials took place in occupied huts, replicating natural climate and typical real-world use. Scientific evaluation and experimental validation of community claims of the effectiveness of plants as repellents is important in promoting and preserving the use of effective species as well as in discouraging the use of non-effective species [50,58]. Mosquito catches during the plant-smoke repellent trials were low across all *Anopheles* species, even though sampling was timed for the study area’s peak malaria-transmission period. The NMCP has a continuous ITN distribution programme in the study area and that likely reduces the populations of the malaria vectors. Small sample sizes for *An. gambiae* s.l. during knockdown tests and for *An. gambiae* s.l., *An. rufipes* and *An. coustani* during field repellent efficacy tests reduced the reliability and reproducibility of the results, making it difficult to draw definitive species-specific conclusions. Small sample sizes result in lower statistical power, increases the probability of a false negative and overestimate a true effect [59]. A true negative control group without any type of smoke was necessary to assist in establishing a real baseline for comparing observed effects. Future studies should consider testing plant smoke repellents against a gold-standard repellent, like DEET, to establish a benchmark for performance under local field conditions.

The field study was conducted in an area with low vector densities and the results may have limited applicability to areas with high vector populations and intense malaria transmission. This is because plant smoke that reduces mosquito bites at low densities may not be sufficient to bring malaria transmission below the threshold necessary for disease prevention in high transmission settings. High vector densities may overwhelm the plant smoke repellent effect through an increase in overall biting pressure. In addition, different malaria vectors have different biting behaviours and responses to repellent such that studies conducted on a low malaria vector density of one species may not reflect the biting behaviour of other vectors present in a high transmission setting [28]. To understand plant smoke repellent limitations and improve its applicability to high-transmission settings, future studies should incorporate dose-response testing (testing repellents under various malaria vector density conditions), focus on epidemiological endpoints (disease transmission rates) and consider human behaviour (willingness of study participants to use a plant smoke repellent in the presence of high density of vectors).

The knockdown effect of a mosquito repellent product reflects its ability to minimize human-mosquito contact or biting chances [50]. There were no knocked-down mosquitoes recorded during the 150-minute exposure period in the control (charcoal) group indicating that smoke or heat produced from the burning charcoal had no toxic effect on *An. gambiae* s.l. The 100% knockdown of *An. gambiae* s.l. achieved by smoke from thermal expulsion of *V. payos* and *P. africanum* during the 150-minute exposure period from the present study compared well with those of *Cananga odorata* on *Anopheles dirus* [60]; and those of *Tagetes erecta*, *T. minuta* and *Lippia javanica* on *An. arabiensis* [50]. *Adansonia digitata* smoke failed to achieve 100% knockdown in *An. gambiae* s.l. during the 150-minute exposure time and this can be attributed to a short exposure period or to the composition of its smoke that is dependent on the nature of the phytochemical constituents of the plant. It is possible that the plant had reached its knock down peak and it could not inflict any further knock down in the *An. gambiae* s.l. mosquitoes during the exposure period. Similar results were reported for *Lantana camara* against *An. arabiensis* [50]. Longer exposure periods or mixed plant formulations may increase the knockdown effect of *P. africanum* [50,61]*.*

Smoke produced from thermal expulsion of *V. payos* had the fastest knockdown rates when compared to smoke from thermal expulsion of *P. africanum* and *A. digitata*. The kdT₅₀ for *V. payos* was 37.9 min (95% CI 32–44) versus 44.75 (95% CI 41.60–47.79) for *P. africanum* and 77.8 min (95% CI 68.02–87.49) for *A. digitata*. Similarly, the kdT_90_ for *V. payos* was 69.74 min (95% CI 59.97–86.04) versus 89.66 min (95% CI 83.18–97.85) for *P. africanum* and 146.95 min (95% CI 138.20–170.11) for *A. digitata*. However, the kdT_50_ and kdT_90_ rates for *V. payos* from the present study were slower than that for *Eucalyptus citriodora*, *E. tereticornis* and *Chenopodium ambrosioides on An. gambiae* [62] or that for *T. erecta*, *T. minuta* and *E. grandis* on *An. arabiensis* [50]. The use of plants with a fast knock down effect to repel mosquitoes is encouraged since an effective repellent prevents human-vector contact and this is important in reducing disease transmission. Smoke produced from thermal expulsion of *V. payos*, achieved more than 90% percent 24-hr mortality used as the minimum criteria for insecticidal effect [56]. *Peltophorum africanum* and *A. digitata* smoke fell below the EPA [56] 90% 24-hr mortality and showed moderate insecticidal effect. Results for *P. africanum* and *A. digitata* are similar to that for smoke produced from burning *Cymbopogon nardus* (citronella), *Satureja hortensis* (savory), and *Thymus vulgaris* (thyme) [29], *E. citriodora*, *E. tereticornis* and *C. ambrosioides* [62], and *T. minuta* and *L. javanica* [50]. Results from knockdown and 24-hr mortality of *An. gambiae* s.l. obtained from present study are important, as they indicate that smoke produced from thermal expulsion of *V. payos* leaves is effective at reducing *An. gambiae* s.l. densities and this may lead to a reduction in disease transmission. Hence, smoke produced from burning *V. payos* have potential as a complementary tool to the major vector control methods (IRS and ITNs) and may have a significant role in integrated vector management (IVM) programs in the study area.

An effective repellent reduces man-vector contact and interrupts disease transmission [63]. Smoke from thermal expulsion of *V. payos* met the EPA [56] 75% minimum standard for spatial repellency (landing-rate suppression) against all the mosquito species. *Adansonia digitata* met the minimum standard for repellency for *An. gambiae* s.l. and *Culex* spp. Although, *P. africanum* significantly reduced both *An. funestus* s.l. and *Culex* spp catches during repellent tests, it failed to meet the 75% minimum performance standard for spatial repellency [56]. While this discrepancy highlights an important difference between statistical significance/relevance and practical applicability, future research studies should confirm the repellent efficacy of *P. africanum* against different mosquito species under scenarios with different mosquito densities. The repellency efficacy of both *P. africanum* and *A. digitata* can be improved by using mixed plant formulations. Mixtures enhance the repellency and insecticidal efficacy of repellent plants because a holistic formulation of plants has additive or synergistic activities that each plant lacks when used in isolation [61,62]. The results from the present study were comparable to those obtained from burning *Thymus serpyllum, Daniella oliver* and *Hyptis suaveolens* [19]. Similar results were also obtained using *Corymbia citriodora* against *An. gambiae* in Kenya [30], and using some aromatic plants against *An. arabiensis* and *An. pharoensis* in Ethiopia [32]. However, the percentage reduction in mosquito biting due to smoke produced from thermal expulsion of native plants investigated in the present study were lower than that of smoke produced from thermal expulsion of mixed powders from *Azadirachta indica*, *Eucalyptus camaldulensis* and *Ociumum forskolin* [33]. The mixed powders from *A. indica*, *E. camaldulensis* and *O. forskolin* reduced *An. arabiensis* and *Ae aegypti* biting by 94% and 91% respectively in field studies in Ethiopia.

In the present study, only *V. payos* achieved 92% 24-hr mortality for *An. gambiae* s.l. and 86% reduction in biting for *An. funestus* s.l., suggesting eligibility for scale-up trials. The performances of *P. africanum* and *A. digitata* were below the EPA [56] minimum performance standards for spatial repellents, suggesting that *P. africanum* and *A. digitata* should be used with caution. The differences in the performances of the three plants are consistent with those from plants evaluated in previous studies [19,30–33]. The differences can be explained by taking into account the differences in plant species tested and mosquito species involved. The variation in efficacy depending on plant and mosquito species represents a biologically important finding that suggests the need for specific and personalized approaches in developing vector control strategies using native plants.

The mosquito species and plant smoke-specific percentage reduction in biting found in the present study implies that the results for a specific mosquito species cannot be extrapolated to other mosquito species. Furthermore, the present study was carried out in Bare ward, Mazowe district, Zimbabwe, during the post-rainfall season and the findings might not be extrapolated to different ecological contexts or seasons in the country or elsewhere in Africa where the three plants are found. Malaria vector behaviour and epidemiology vary across geographical settings [5], and this makes it difficult for the relationship between malaria vectors and smoke from plants found in one part of the country to have global applicability. Consequently, the results from the present study could be location-specific and their applicability to other settings maybe limited. Several factors are responsible for the differences in percent repellency recorded in the present study. Firstly, this could be a result of mosquito species-dependent sensitivity to smoke produced from burning different plants. Different mosquito species behave or respond differently to a given chemical cue [28]. Secondly, there is great diversity in the chemical constituents of plants, which vary with plant species, variety and between populations and geographical locations within the same variety [34,64]. The quality, quantity, and composition of bioactive phytochemical compounds found in plants as well as their repellent and insecticidal properties are influenced by genetic, ontogenic (age and stage of development or vegetative cycle stage, organ of plant) and environmental (soil type and design, altitude, climate and biotic) factors [64,65].

Overall, smoke from thermal expulsion of native plants investigated in the present study was effective against both *Anopheles* and *Culex* mosquitoes. Although, the exact mechanism of action of smoke produced from burning repellent plants in preventing mosquito bites is not fully understood [66], the traditional method of using smoke from burning plant materials to repel mosquitoes investigated in the present study proved to be a simple and effective method to reduce human-mosquito contact. The traditional smoking of rooms using effective native repellent plants can be included as one of the IVM strategies to make vector control more affordable, effective and sustainable for the disadvantaged and marginalized communities. Economically, using native plants for mosquito control is relatively cheaper than commercial synthetic repellents. The plant parts used, especially leaves, are easy to harvest and store. *Adansonia digitata* fruit shells are a freely available waste product that can benefit even the poorest members of the community, and can supplement ITNs in Bare ward in malaria control intervention, particularly during the early part of the evening before bedtime.

Despite the effectiveness of traditional repellent plants against mosquito bites, there are some safety concerns such as respiratory discomfort, allergic reactions and eye irritation that are related to household air pollution and the use of smoke as a mosquito repellent [67]. Household air pollution due to biomass smoke is a global health problem [68]. The health risks posed by household air pollution are widely accepted with approximately 3.2 million deaths from chronic respiratory diseases such as chronic obstructive pulmonary disease and acute respiratory infections, attributed to household air pollution, recorded worldwide in 2020 [68]. Exposure to household air pollution also increases the risks of cardiovascular diseases such as ischemic heart disease and stroke. Women and children under five are disproportionately affected by household air pollution in low- and middle-income countries where limited access to clean energy, particularly in rural areas, lead to the widespread use of biomass fuels such as wood, dung, crop residues and charcoal that are readily available and affordable [68]. A short exposure period (4 hours) in the present study could have reduced the carbon monoxide or particulate exposure risk for occupants but there is need to evaluate the safety of smoke produced from burning powders from *V. payos*, *P. africanum* and *A. digitata* before a low-cost high-impact plant-based household protection tool can be developed from the plants.

While smoke from burning traditional repellent plants can be effective, it may not be practical for daily use due to factors related to the short duration of smoke effects that might require frequent reapplication, and inconveniences related to the need for constant monitoring, proper ventilation and safe space for burning the plants. Future studies should consider standardizing plant extracts and formulating them into topical oils or sprays that provide longer-lasting protection, enhancing the practicality and efficacy of traditional repellent plants. Local healers provide valuable insights into traditional practices and plant uses and collaborating with them fosters community trust and ownership of mosquito control efforts, facilitating the development of effective, culturally acceptable/relevant repellents.

The key to future plant-based mosquito repellent products in the country may depend on the untapped potential of traditional repellent plants. The protection of traditional knowledge and intellectual property rights of indigenous communities requires a multifaceted approach and is crucial for preserving cultural heritage and promoting equitable benefit sharing. The availability of *V. payos*, *P. africanum* and *A. digitata* for use in mosquito control programs is almost guaranteed; these plants can meet the demands of the local communities since they are naturally adapted to local soils and climates; and as native species, they can adapt and survive environmental stresses better than alien species. However, their potential for higher yields and growth is limited since they are currently only growing in the wild. Illegal and indiscriminate use of the plants for various purposes might also be a challenge. Further exploration of the potential of these plants as vector control agents in a holistic manner, including their domestication, processing and commercialization is required and significant. This includes the evaluation of the effect of smoke from the mixtures of the plants in improving knockdown rates and in reducing biting by malaria vectors.

User acceptance and compliance are important for any vector control tool to achieve optimum effectiveness. Community members in the study area are familiar with the use of plant smoke to repel mosquitoes with burning or smouldering as the main modality of applying the traditional repellent plants [27]. The households involved expressed interest in adopting the plant repellents in the tested form and appreciated the effectiveness, accessibility and cultural significance of this intervention, which resembled their own practices and connected them to their heritage and traditions. They also expressed willingness to conserve or grow and harvest the plants themselves, making this intervention a valuable community-based solution for localized mosquito control. However, to improve the community initiative, thermal expulsion of selected mosquito-repellent plants was adopted in place of direct burning in the present study. Thermal expulsion is a simple technique that is culturally acceptable since the amount of smoke produced is reduced, it is highly adaptable and yields higher levels of repellency for several plant species than direct burning [9]. Thermal expulsion of effective native plants may encourage user compliance among the rural communities in malaria-endemic regions of the country due to its traditional nature, low cost and easy accessibility. In addition, the traditional charcoal “*mbaura*” stoves used in the present study have a long history of use for cooking and heating in rural communities in Zimbabwe. These traditional charcoal stoves may provide a relatively cheap means for the continuous production of smoke from thermal expulsion of repellent plants prior to bedtime when protection from mosquito bites is mostly required, benefitting low-income households. However, burning charcoal can pose a fire risk and it is essential that thermal expulsion be done safely in controlled environments and this can be achieved through community dialogue and education.

Dried and ground plant materials were used in the present study since previous investigations had revealed that smoke from burning dried samples were more repellent than that of fresh samples [69]. The drying and laboratory processing of plant samples steps described in the present study are not easily replicable in community settings because of the costly nature of the laboratory mill/electric grinder that also requires electricity. However, communities can dry plant samples in the shade in their communities and alternatively use a traditional mortar and pestle to process the dried plant materials into fine powders. Although the use of the mortar and pestle is more affordable, self-sufficient and sustainable than the laboratory mill/electric grinder, it has limitations related to standardization, scalability and quality control. The use of the mortar and pestle may not be suitable for large-scale production of plant powders for mosquito control and ensuring consistent quality of plant powders using this simple technique or traditional alternative can be challenging.

Wild populations of *An. gambiae* s.l. used in the knockdown experiments represent better the response of field populations in their natural environment [51]. Despite the predominance of wild free flying adults of *An. funestus* s.l. in repellent tests, the study failed to raise this species from larval collections and had to rely on *An. gambiae* s.l. only for the knockdown tests. Although field repellent tests demonstrated differential efficacy of tested plants, the differences in species composition between knockdown tests and repellency tests compromised direct comparability of results and the extrapolation and relevance of knockdown findings for real field conditions. Different mosquito species, including different populations of the same species, can have varying sensitivities to repellents [28], suggesting that efficacy results may differ significantly depending on target species.

CDC light traps used as a surrogate for estimating repellency in this study reduce the risk of exposing study participants to disease vectors and they do not have the ethical constraints that are associated with human landing collections (HLC) [9]. CDC light traps set beside occupied untreated nets sample similar or equivalent host-seeking cohorts and age distribution of *An. gambiae* s.l. and *An. funestus* s.l. populations as HLC [9,70]. In addition, CDC light traps are relatively easier and less labour-intensive than HLC [9,53]. However, CDC light traps underestimate the actual biting risk [53]. In addition, competing light sources, including moonlight and house lights negatively affect the performance of CDC light traps while the batteries need frequent recharging.

This study noted three major limitations that constrain the interpretation of results or generalization of the study findings. Firstly, due to logistical constraints, only three native plant species were investigated. This is a small fraction of the repellent plants traditionally used by communities in Zimbabwe. The focus on only three plant species provides an incomplete picture of traditional repellent plants in the study area and may have overlooked other potentially more effective ones. The repellent properties of plants can be highly variable, even within the same family, due to differences in their chemical composition [6,7]. In addition, the repellent efficacy of a plant can vary depending on the part of the plant used, the plant’s age and the method of preparation [6,7]. Secondly, the study could not identify the sibling species of the *An. gambiae* and *An. funestus* complexes and their insecticide resistance status due to logistical constraints. Different mosquito species, including different populations of the same species, can have varying sensitivities to repellents [28]. Plant smoke may not repel the actual vector of concern such that without sibling species identification, it is impossible to indicate the particular mosquito species being repelled, leading to ineffective vector control or making results difficult to interpret and replicate. In addition, some malaria vectors may develop behavioural adaptations to plant-based repellents, and it would be impossible to determine if a particular plant smoke is effective against these resistant populations without sibling species identification. Thirdly, the safety of repellent plants’ smoke to the occupants and its effect on non-target organisms and the environment were not investigated as this was beyond the scope of the present study. Traditional use of plant smoke does not guarantee safety and burning plant materials without toxicological analysis may pose health risks upon inhalation or dermal contact. Plant smoke can adversely affect biodiversity by harming beneficial insects and disrupting local ecosystems. In addition, the lack of standardization and toxicity data on plant smoke makes it difficult to develop and commercialize safe, consistent and effective repellents. Other limitations affected both reliability and the capacity to generalize findings to other populations and context. For instance, although the “*mbaura”* stoves with burning plant material were placed at the centre of each hut, uniform smoke exposure throughout all parts of the huts was not guaranteed during the experiments and there was no control of wind that affects smoke dispersion. Furthermore, mosquito counting by different researchers during the experiments introduced observer bias in results. Finally, the present study did not evaluate how long protection from plant smoke was sustained because it was slightly beyond its scope, however future studies should consider collecting this data as it enhances the practical relevance of plant smoke in mosquito control.

## Conclusions

Results from the present study indicated that thermal expulsion of effective traditional repellent plants is a simple, low technology, locally adaptable and culturally acceptable technique that is effective at repelling, knocking down, and killing *Anopheles* and *Culex* mosquitoes. Smoke from burning effective traditional repellent plants can be adopted as a low-cost high-impact alternative to synthetic repellents in combination with major vector control measures such as ITNs, and IRS to control malaria. Studies to determine whether the use of smoke from burning effective traditional repellent plants has a community effect that can lead to the reduction of malaria burden are needed. Ethnobotanical data is critical for understanding traditional practices and identifying potential repellent plants for research. Future studies should consider testing a wider range of plant species and plant parts, along with different methods of preparations or applications and traditional repellent plants used as poultices or rubbed on the skin can be prioritized. Molecular techniques should be employed to identify sibling species of wild free-flying malaria vectors, while safety evaluations and toxicological assessments should be conducted for plant smoke inhalation and skin exposure in future studies. It is also crucial to assess the potential impact of smoke on beneficial insects and local ecosystems in future studies. Finally, field studies should test plant products under different malaria transmission conditions to ensure reliable, consistent and comparable results.

## References

[1] Sande S, Zimba M, Chinwada P, Masendu HT, Makuwaza A. Malaria vector species composition and relative abundance in Mutare and Mutasa districts, Zimbabwe. J Entomol Acarol Res. 2015;4:4955.

[2] Masendu HT, Hunt RH, Koekemoer LL, Brooke BD. Spatial and temporal distributions and insecticide susceptibility of malaria vectors in Zimbabwe. African Entomol. 2005;13(1):25–34.

[3] Sande S, Zimba M, Chinwada P, Masendu HT, Makuwaza A. Biting behaviour of Anopheles funestus populations in Mutare and Mutasa districts, Manicaland province, Zimbabwe: Implications for the malaria control programme. J Vector Borne Dis. 2016;53(2):118–26. doi: 10.4103/0972-9062.184831 27353581

[4] Killeen GF, Govella NJ, Lwetoijera DW, Okumu FO. Most outdoor malaria transmission by behaviourally-resistant Anopheles arabiensis is mediated by mosquitoes that have previously been inside houses. Malar J. 2016;15:225. doi: 10.1186/s12936-016-1280-z 27093890 PMC4837512

[5] World Health Organization. World Malaria Report. 2022. Available from: file:///C:/Users/OAI/Downloads/9789240064898-eng.pdf

[6] Maia MF, Moore SJ. Plant-based insect repellents: a review of their efficacy, development and testing. Malar J. 2011;10(Suppl 1):S11. doi: 10.1186/1475-2875-10-S1-S11 21411012 PMC3059459

[7] Bekele D. Review on insecticidal and repellent activity of plant products for malaria mosquito control. Biomed Res Rev. 2018;2(2):1–7.

[8] Moore EL, Scott MA, Rodriguez SD, Mitra S, Vulcan J, Cordova JJ, et al. An online survey of personal mosquito-repellent strategies. PeerJ. 2018;6:e5151. doi: 10.7717/peerj.5151 30002979 PMC6034598

[9] Seyoum A, Killeen GF, Kabiru EW, Knols BGJ, Hassanali A. Field efficacy of thermally expelled or live potted repellent plants against African malaria vectors in western Kenya. Trop Med Int Health. 2003;8(11):1005–11. doi: 10.1046/j.1360-2276.2003.01125.x 14629767

[10] Nguyen Q-BD, Vu M-AN, Hebert AA. Insect repellents: An updated review for the clinician. J Am Acad Dermatol. 2023;88(1):123–30. doi: 10.1016/j.jaad.2018.10.053 30395919

[11] Karunamoorthi K, Hailu T. Insect repellent plants traditional usage practices in the Ethiopian malaria epidemic-prone setting: an ethnobotanical survey. J Ethnobiol Ethnomed. 2014;10:22. doi: 10.1186/1746-4269-10-22 24521138 PMC3932844

[12] Benelli G, Mehlhorn H. Declining malaria, rising of dengue and Zika virus: insights for mosquito vector control. Parasitol Res. 2016;115(5):1747–54. doi: 10.1007/s00436-016-4971-z 26932263

[13] Gou Y, Li Z, Fan R, Guo C, Wang L, Sun H, et al. Ethnobotanical survey and evaluation of traditional mosquito repellent plants of Dai people in Xishuangbanna, Yunnan Province, China. J Ethnopharmacol. 2020;262:113124. doi: 10.1016/j.jep.2020.113124 32730874

[14] Diaz JH. Chemical and Plant-Based Insect Repellents: Efficacy, Safety, and Toxicity. Wilderness Environ Med. 2016;27(1):153–63. doi: 10.1016/j.wem.2015.11.007 26827259

[15] Leal WS. The enigmatic reception of DEET - the gold standard of insect repellents. Curr Opin Insect Sci. 2014;6:93–8. doi: 10.1016/j.cois.2014.10.007 25530943 PMC4269249

[16] Ladan Z, Okoli B, Mtunzi F. Efficacy and Safety of Essential Oils in The Control of Mosquito: A Review of Research Findings. Int J Chem. 2022;14(2):45–58. doi: 10.5539/ijc.v14n2p45

[17] Curtis CF, Line JD, Baolin I, Renz A. Natural and synthetic repellents. In: Curtis CF, editor. Appropriate Technology in Vector Control. London: Wolfe; 1989.

[18] Bonkian LN, Yerbanga RS, Coulibaly MT, Levevre T, Sangare I, Ouédraogo T, et al. Plants against malaria and mosquitoes in Sahel region of Burkina Faso: An ethnobotanical survey. Int J Herb Med. 2017;5(3):82–7.

[19] Pålsson K, Jaenson TG. Plant products used as mosquito repellents in Guinea Bissau, West Africa. Acta Trop. 1999;72(1):39–52. doi: 10.1016/s0001-706x(98)00083-7 9924960

[20] Kweka EJ, Mosha F, Lowassa A, Mahande AM, Kitau J, Matowo J, et al. Ethnobotanical study of some of mosquito repellent plants in north-eastern Tanzania. Malar J. 2008;7:152. doi: 10.1186/1475-2875-7-152 18687119 PMC2519077

[21] Karunamoorthi K, Ilango K, Endale A. Ethnobotanical survey of knowledge and usage custom of traditional insect/mosquito repellent plants among the Ethiopian Oromo ethnic group. J Ethnopharmacol. 2009;3:414. 10.1016/j.jep.2009.07.008 19607902

[22] Mavundza EJ, Maharaj R, Finnie JF, Kabera G, Van Staden J. An ethnobotanical survey of mosquito repellent plants in uMkhanyakude district, KwaZulu-Natal province, South Africa. J Ethnopharmacol. 2011;137(3):1516–20. doi: 10.1016/j.jep.2011.08.040 21893183

[23] Kantheti P, Padma A. Ethnobotanical tribal practices for mosquito repellency followed by the people of north India. J Pharmacol Phytochem. 2017;6(6):942–4.

[24] Youmsi RDF, Fokou PVT, Menkem EZ, Bakarnga-Via I, Keumoe R, Nana V, et al. Ethnobotanical survey of medicinal plants used as insects repellents in six malaria endemic localities of Cameroon. J Ethnobiol Ethnomed. 2017;13(1):33. doi: 10.1186/s13002-017-0155-x 28595645 PMC5465592

[25] Ejecta D. Ethno-botanical survey of plants used for prevention against mosquito bites and control of malaria in Assosa district, western Ethiopia. Int J Ethnobiol Ethnomed. 2019;1:1–9.

[26] Lukwa N, Nyazema NZ, Curtis CF, Mwaiko GL, Chandiwana SK. People’s perceptions about malaria transmission and control using mosquito repellent plants in a locality in Zimbabwe. Cent Afr J Med. 1999;45(3):64–8. doi: 10.4314/cajm.v45i3.8456 10565064

[27] Nyasvisvo DS, Nhiwatiwa T, Sithole R, Sande S, Chapano C. An ethnobotanical survey of plants used against host-seeking mosquitoes by communities in Mazowe and Shamva districts, Zimbabwe. Ethnobot Res and App. 2024;28:1–19.

[28] Moore SJ, Lenglet A, Hill N. Plant-based insect repellents. In: Insect repellents: principles methods and use. Boca Raton, Florida: CRC Press; 2006.

[29] Paru R, Hii J, Lewis D, Alpers MP. Relative repellency of woodsmoke and topical applications of plant products against mosquitoes. PNG Med J. 1995;38(3):215–21. 9522861

[30] Seyoum A, Kabiru EW, Lwande W, Killeen GF, Hassanali A, Knols BGJ. Repellency of live potted plants against Anopheles gambiae from human baits in semi-field experimental huts. Am J Trop Med Hyg. 2002;67(2):191–5. doi: 10.4269/ajtmh.2002.67.191 12389946

[31] Waka M, Hopkins R, Curtis C. Ethnobotanical survey and testing of repellent plants traditionally used in Ethiopia. J Ethnopharmacol. 2004;95:95–101. 15374613 10.1016/j.jep.2004.07.003

[32] Dugassa S, Medhin G, Balkew M, Seyoum A, Gebre-Michael T. Field investigation on the repellent activity of some aromatic plants by traditional means against Anopheles arabiensis and An. pharoensis (Diptera: Culicidae) around Koka, central Ethiopia. Acta Trop. 2009;112(1):38–42. doi: 10.1016/j.actatropica.2009.06.002 19539591

[33] Wendimu A, Tekalign W. Field efficacy of ethnomedicinal plant smoke repellency against Anopheles arabiensis and Aedes aegypti. Heliyon. 2021;7(7):e07373. doi: 10.1016/j.heliyon.2021.e07373 34258465 PMC8258845

[34] Nyasvisvo DS, Sithole R, Sande S, Nhiwatiwa T. GC-MS screening of phytochemicals in three plants traditionally used as mosquito repellents in Mazowe district, Zimbabwe. Arab J Med Aromat Plants. 2025;11(2):45–77.

[35] Zimbabwe National Statistical Agency. Population and Housing Census 2022 National Report. Harare, Zimbabwe. 2022. Available from: http://www.zimstat.co.zw/

[36] Nyasvisvo DS, Nhiwatiwa T, Sithole R, Sande S. Characterization of Anopheles mosquito breeding habitats for malaria vector control in Mazowe and Shamva districts, Zimbabwe. J Vector Borne Dis. 2025;62(2):154–64. doi: 10.4103/JVBD.JVBD_85_24 39373225

[37] Bradley K, Ingham K. Zimbabwe. Encyclopedia Britannica. 2020. Available from: https://www.britannica.com/place/Zimbabwe

[38] USAID President’s Malaria Initiative. Zimbabwe Malaria Profile. FY 2024 Zimbabwe Country Profile. President’s Malaria Initiative. 2023. Available from: https://www.pmi.gov/wp-content/uploads/2024/04/FY-2024-Zimbabwe-Country-Profile.pdf

[39] Chapano C, Mamuto M. Plants of the Chimanimani district. National Herbarium and Botanic Garden; 2003.

[40] Maroyi A. Traditional use of medicinal plants in south-central Zimbabwe: review and perspectives. J Ethnobiol Ethnomed. 2013;9:31. doi: 10.1186/1746-4269-9-31 23642285 PMC3653698

[41] Nyamoita MG. Anti-larval compounds from Vitex schiliebeni and Vitex payos. MSc Thesis. Kenyatta University, Nairobi. Kenya; 2011.

[42] Nyamoita M, Mbwambo Z, Ochola B, Innocent E, Lwande W, Hassanali A. Chemical composition and evaluation of mosquito larvicidal activity of Vitex payos extracts against Anopheles gambiae Giles S.S larvae. Spatula DD. 2013;3(3):113. doi: 10.5455/spatula.20130806054707

[43] Chapano C, Mugarisanwa NH. Plants of the Matopo District. National Herbarium and Botanic Garden; 2003.

[44] Maroyi A. An ethnobotanical survey of medicinal plants used by the people in Nhema communal area, Zimbabwe. J Ethnopharmacol. 2011;136(2):347–54. doi: 10.1016/j.jep.2011.05.003 21575701

[45] Theo A, Masebe T, Suzuki Y, Kikuchi H, Wada S, Obi CL, et al. Peltophorum africanum, a traditional South African medicinal plant, contains an anti HIV-1 constituent, betulinic acid. Tohoku J Exp Med. 2009;217(2):93–9. doi: 10.1620/tjem.217.93 19212101

[46] Moyo S, Gwitira I, Murwira A, Zengeya FM, Shekede MD. Spatial distribution and abundance of the African baobab (Adansonia digitata) in Zimbabwe. Trans R Soc S Afr. 2019;74(3):213–8.

[47] Besco E, Braccioli E, Vertuani S, Ziosi P, Brazzo F, Bruni R, et al. The use of photochemiluminescence for the measurement of the integral antioxidant capacity of baobab products. Food Chem. 2007;102(4):1352–6. doi: 10.1016/j.foodchem.2006.05.067

[48] Das S, Garver L, Dimopoulus G. Protocol for rearing mosquitoes (Anopheles gambiae). J Visual Exp. 2007;5:221–5. 10.3791/221 PMC2557088 18979019

[49] Coetzee M. Key to the females of Afrotropical Anopheles mosquitoes (Diptera: Culicidae). Malar J. 2020;19(1):70. doi: 10.1186/s12936-020-3144-9 32054502 PMC7020601

[50] Lukwa N, Mduluza T, Nyoni C, Lukwa AT, Zimba M. Knockdown and insecticidal activity of the plants Tagetes minuta, Lippia javanica, Lantana camara, Tagetes erecta, and Eucalyptus grandis on Anopheles arabiensis mosquitoes. J Entomol Acarol Res. 2018;50:7241.

[51] World Health Organization. Guidelines for efficacy testing of spatial repellents. 2013.

[52] Dafaallah AB. Fundamentals of design and analysis of agricultural experiments (Observation-Experimentation-Discussion). Part Two First Edition. University of Gezira House for Printing and Publishing, Wad Medani, Sudan. 2017.

[53] Lines J, Curtis C, Wilkes T, Njunwa K. Monitoring human-biting mosquitoes (Diptera: Culicidae) in Tanzania with light traps hung beside mosquito nets. Bull Entomol Res. 1991;81:77–84.

[54] Mboera LEG. Sampling techniques for adult Afrotropical malaria vectors and their reliability in the estimation of entomological inoculation rate. Tanzan Health Res Bull. 2005;7(3):117–24. doi: 10.4314/thrb.v7i3.14248 16941936

[55] Finney DJ. Probit Analysis: A Statistical Treatment of the Sigmoid Response Curve. Cambridge: Cambridge University Press; 1952.

[56] -224Environmental Protection Agency. Pesticide product performance data requirements for products claiming efficacy against certain invertebrate pests. Environ Protect Agency Federal Reg. 2022;87(73):22464–84.

[57] Macdonald R. Using Written Consent Forms When Conducting Non-Elite Qualitative Research: Reflections from Zambia. J South African Stud. 2024;50(2):195–206. doi: 10.1080/03057070.2024.2386650

[58] Lalthazuali MN. Mosquito repellent activity of volatile oils from selected aromatic plants. Parasitol Res. 2017;116(2):821–5. doi: 10.1007/s00436-016-5351-4 28013374

[59] Biau DJ, Kernéis S, Porcher R. Statistics in brief: the importance of sample size in the planning and interpretation of medical research. Clin Orthop Relat Res. 2008;466(9):2282–8. doi: 10.1007/s11999-008-0346-9 18566874 PMC2493004

[60] Soonwera M. Efficacy of essential oil from Cananga odorata (Lank.) Hook.f. & Thomson (Annonaceae) against three mosquito species Aedes aegypti (L.), Anopheles dirus (Peyton and Harrison), and Culex quinguefasciatus (Say). Parasitol Res. 2015;114:4531–43. 26337270 10.1007/s00436-015-4699-1

[61] Bussmann RW, Sharon D. Traditional medicinal plant use in Northern Peru: tracking two thousand years of healing culture. J Ethnobiol Ethnomed. 2006;2:47. doi: 10.1186/1746-4269-2-47 17090303 PMC1637095

[62] Bossou AD, Mangelinckx S, Yedomonhan H, Boko PM, Akogbeto MC, De Kimpe N, et al. Chemical composition and insecticidal activity of plant essential oils from Benin against Anopheles gambiae (Giles). Parasit Vectors. 2013;6:337. doi: 10.1186/1756-3305-6-337 24298981 PMC3866997

[63] Kazembe T, Chaibva M, Nkomo S. Evaluation of mosquito repellence of Ocimum americanum and Blumea alata plants. J Sci Res Pharm. 2012;1(2):55–7.

[64] Debella A. Manual for phytochemical screening of medicinal plants. Addis Ababa, Ethiopia: EHNRI; 2002.

[65] Regnault-Roger C, Vincent C, Arnason JT. Essential oils in insect control: low-risk products in a high-stakes world. Annu Rev Entomol. 2012;57:405–24. doi: 10.1146/annurev-ento-120710-100554 21942843

[66] Nyawira Wangai L, Kimani Kamau K, Munyekenye G, Nderu D, Maina E, Gitau W, et al. Efficacy of Plant-based Repellents Against Anopheles Mosquitoes: A Systematic Review. Biomed Sci. 2020;6(3):44. doi: 10.11648/j.bs.20200603.11

[67] Vernède R, van Meer MM, Alpers MP. Smoke as a form of personal protection against mosquitos, a field study in Papua New Guinea. Southeast Asian J Trop Med Public Health. 1994;25(4):771–5. 7667730

[68] World Health Organization. Household air pollution. 2023.

[69] Dube FF, Tadesse K, Birgersson G, Seyoum E, Tekie H, Ignell R et al. Fresh, dried or smoked? Repellent properties of volatiles emitted from ethnobotanical plant leaves against malaria and yellow fever vectors in Ethiopia. Malar J. 2011;10:375. 22182798 10.1186/1475-2875-10-375 PMC3285543

[70] Magbity EB, Magbity EB, Lines JD, Marbiah MT, David K, Peterson E. How reliable are light traps in estimating biting rates of adult Anopheles gambiae s.l. (Diptera: Culicidae) in the presence of treated bed nets? Bull Entomol Res. 2002;92(1):71–6. doi: 10.1079/BER2001131 12020364

